# Implementation of fireworks-related injury surveillance in Metro Manila, Philippines, 2023–2024

**DOI:** 10.5365/wpsar.2026.17.1.1272

**Published:** 2026-03-16

**Authors:** Kenneth Paul S Ong

**Affiliations:** a Monash University, Melbourne, Victoria, Australia.; b Center for Health Development, Department of Health, Manila, Philippines.

## Abstract

Many countries record high rates of fireworks-related injuries, especially during national celebrations. In the Philippines, increases in the number of injuries reported around the New Year period in recent years have highlighted the importance of continued strengthening of national fireworks-related injury surveillance. The Philippines’ regional epidemiology and surveillance units play a significant role in surveillance by linking its key stakeholders, the sentinel hospitals and the Department of Health’s Central Office. More specifically, these units promote compliance with reporting standards among sentinel hospitals and support case data validation. Approximately half of the sentinel hospitals that contribute data to the surveillance system are in the nation’s capital, Metro Manila. This concentrated coverage prompted the Regional Epidemiology and Surveillance Unit staff in Metro Manila to implement low-cost, digital strategies to improve the quality and timeliness of fireworks-related injury reporting. During the 2023–2024 surveillance period (21 December to 5 January), the use of virtual coordination spaces and data dashboards contributed to reducing turnaround times for generating surveillance reports from 31 minutes to 8 minutes. Moreover, at least 80% of sentinel hospitals provided timely reports on 11 of the 16 days of the surveillance period. Staff commitment was a major contributing factor in overcoming the time and human resource constraints encountered during implementation of these strategies. However, it is important to recognize that beyond these digital innovations, policy reforms that increase funding are needed to enhance fireworks-related injury surveillance and secure its long-term sustainability and scalability in the Philippines.

Fireworks-related injuries (FWRIs) are increasing across the Philippines, following a decline during the first year of the COVID-19 pandemic. (*1*, *2*) Injury prevalence is greatest during December and January, when fireworks are traditionally enjoyed as part of New Year’s celebrations. Injuries rose from 188 cases in December 2021–January 2022 to 291 cases in December 2022–January 2023, an increase of 55%. (*1*, *2*) A further increase was observed the following year, with cases in December 2023–January 2024 roughly doubling the number recorded in December 2022–January 2023. (*3*) Despite policies prohibiting their use, approximately 40% of FWRIs have been attributed to illegal firecrackers. (*1*, *3*) These trends underscore the continuing importance of FWRI surveillance as a basis for targeted interventions to reduce these injuries.

In the Philippines, FWRI surveillance is overseen by the Department of Health (DOH) and runs annually from 21 December to 5 January. (*4*) Throughout this period, the DOH conducts campaigns to remind the public of the dangers associated with fireworks and to promote injury prevention. (*1*, *3*, *5*, *6*) Central to FWRI surveillance is the Online National Electronic Injury Surveillance System (ONEISS), which collates data on identified FWRI cases submitted by a nationwide network of 61 sentinel hospitals using standardized case investigation forms (DOH Department Memorandum 2023–0427). (*3*, *7*) All patients who seek treatment for injuries caused by fireworks at sentinel hospitals are classified as FWRI cases and are further subclassified as fireworks injury, fireworks ingestion, stray bullet injury or fireworks-related tetanus. If no FWRIs are seen on a given day during the surveillance period, sentinel hospitals are required to submit zero reports to ensure completeness of reporting and data integrity (DOH Department Memorandum 2023–0427). The DOH Central Office (CO) reviews and validates all submitted case investigation forms and generates national FWRI reports.

FWRI surveillance is supported by 17 regional epidemiology and surveillance units (RESUs), one for each region of the country. RESUs promote compliance with reporting standards and disseminate FWRI reports to internal and external regional stakeholders. Requests to sentinel hospitals for action on missing, incomplete or unclear data relating to FWRIs are coursed through RESUs.

Almost half of the FWRI sentinel hospitals (*n* = 30) are in Metro Manila, (*4*) making the city a critical focal point for FWRI surveillance reporting and a natural candidate for trialling innovations in surveillance practice. This article documents the operationalization of two digital innovations aimed at improving coordination and reporting efficiency – a virtual coordination space (VCS) and a data dashboard (**Box 1**). Both were implemented by RESU staff in Metro Manila during the 2023–2024 FWRI surveillance period.

**Box 1:**
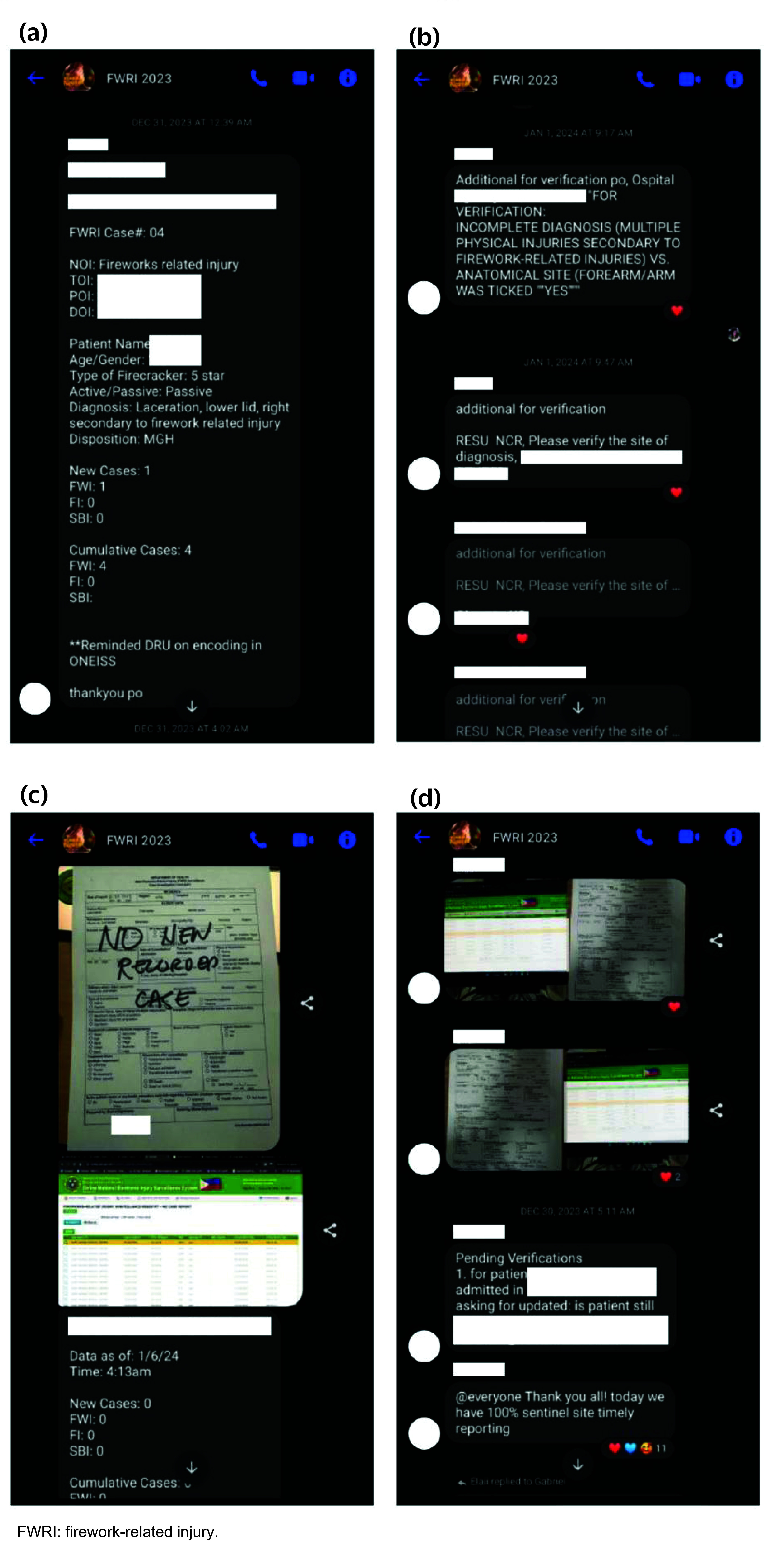
Use of the virtual coordination space: (a) updates on FWRI cases; (b) case validation; (c) zero reporting; and (d) staff feedback

[**insert** Box 1]

## Methods

### Pre-implementation activities

Approximately 3 weeks before the start of the surveillance period, 35 RESU staff were divided into groups of two or three and matched with sentinel hospitals. Due to human resource constraints, some staff were part of more than one group, and some groups were matched with more than one hospital. Two individuals were assigned as co-heads and tasked with liaising directly with the DOH CO and with managing the new e-mail account dedicated to coordinating FWRI surveillance.

After a virtual orientation on liaising with sentinel hospitals, each group gave its assigned sentinel hospital an overview of the FWRI surveillance system and briefed hospital staff on the importance of submitting complete and timely case investigation forms. Hospital staff’s contact details were registered in an online directory. A VCS was created using a free online messaging application, accessible to all involved staff via a mobile phone or computer.

Development of the data dashboard for visualization of FWRI data involved several steps:
consideration of dashboard objectives and its intended users;
design of dashboard elements; and
addition of filters for data customization.

The main objective of the dashboard was to provide the Regional Director of the DOH Metro Manila Center for Health Development and other regional stakeholders with access to the latest FWRI statistics within an hour of the end of each reporting day (06:00 to 05:59 the following day). The dashboard was developed using the free-to-use Google Looker Studio. Looker Studio files were linked to a Google Sheets spreadsheet, into which ONEISS data were uploaded. Data visualizations in the form of tables and graphs were then created using the chart function of Looker Studio. The completed dashboard consisted of the following components:

epidemiologic curve of FWRI cases, by date of injury and comparisons with 2022–2023 data and a 5-year average;
number of FWRI cases by age, sex, local government unit, reporting sentinel hospital, place of injury, patient disposition (treated and sent home, admitted, refused admission) and outcome (alive, died);
anatomic sites affected; and
types of fireworks implicated.

Data filters enabled the generation of granular data by date of injury, local government unit, sentinel hospital and legal status of the implicated fireworks. The ONEISS was cited as the reference for all dashboard visualizations.

### Implementation

**Fig. 1:**
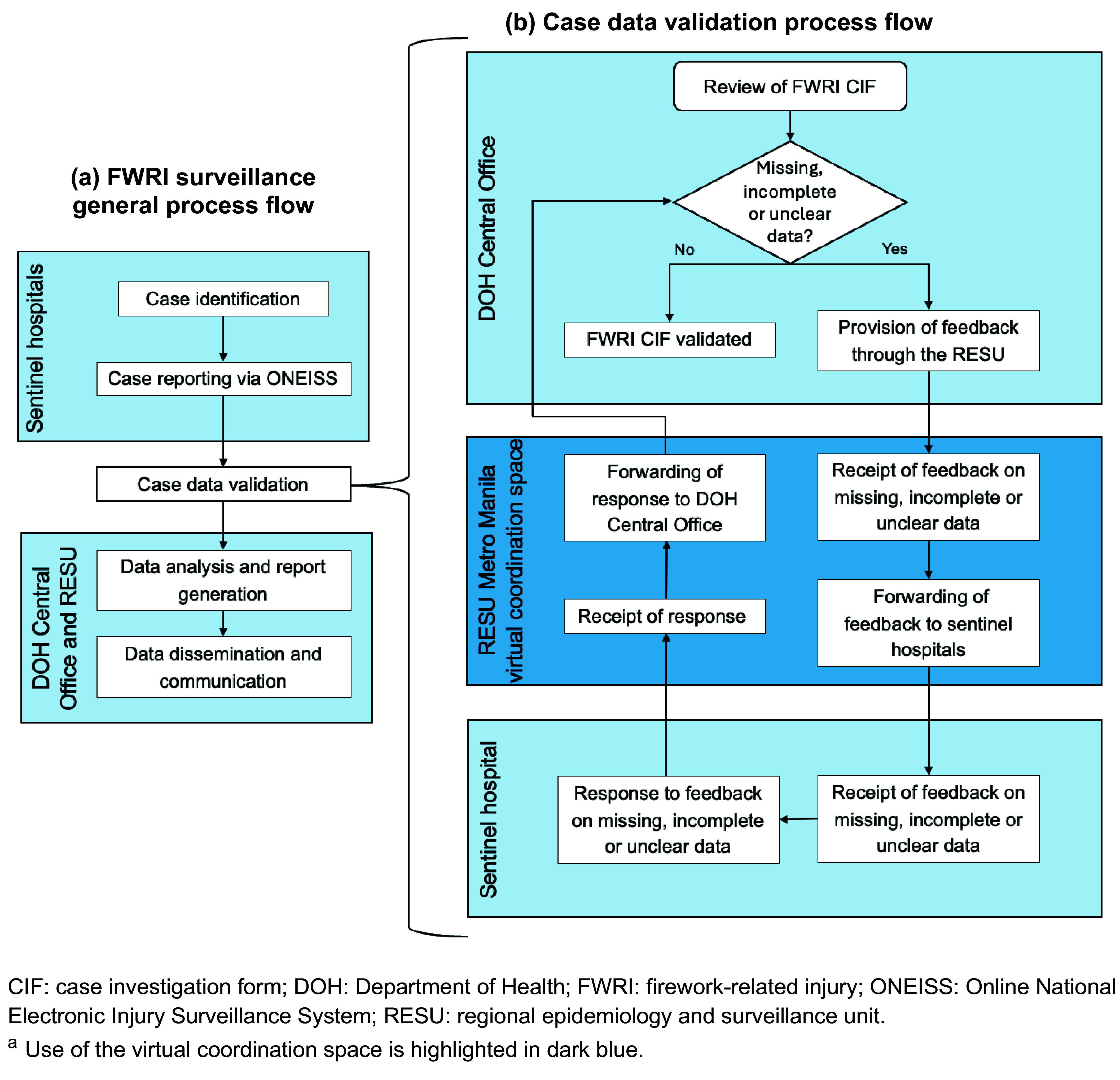
Process flows for FWRI surveillance: (a) general process flow illustrating major activities; and (b) steps for case data validation

Project outputs included an updated online directory of sentinel hospitals, a VCS and a data dashboard. Deliverables included promoting sentinel hospital reporting timeliness; addressing requests for action on missing, incomplete or unclear data; and generating daily FWRI surveillance reports. **Fig. 1** illustrates the process flow for handling requests for action on missing, incomplete or unclear injury data via the VCS as part of data validation. Action requests from the DOH CO were relayed to the co-heads, who posted them in the VCS. RESU staff read the requests and forwarded them to sentinel hospitals. Responses from sentinel hospitals were likewise relayed back through the same mechanism.

[**insert** Figure 1]

At around 02:30, RESU staff issued reminders to sentinel hospitals that had not reported an FWRI for that day to submit a zero report. Proof of hospital compliance with zero reporting was then uploaded to the VCS. Sentinel hospitals that did not submit a zero report were classified as a non-reporting facility. RESU staff were able to contest at the national level any erroneous classification of the facility as non-reporting by taking screenshots of their zero reports.

After the end of each reporting day, RESU staff posted daily and cumulative FWRI statistics for each sentinel hospital in the VCS. In addition, a summary of sentinel hospital timeliness was shared via the VCS, and RESU staff provided sentinel hospitals with appropriate feedback. Sentinel hospitals were considered timely if they submitted FWRI reports on the same day as the date of injury or submitted a zero report before 05:00, whichever criterion applied. Hospitals that did not meet these conditions were classified as late.

To generate daily FWRI surveillance reports, validated case data for Metro Manila were extracted from ONEISS and copied into the online spreadsheet. The dashboard page was refreshed, and the updated report was exported and disseminated to internal and external stakeholders. The link to the dashboard was also shared with stakeholders within the DOH Metro Manila Center for Health Development.

### Post-implementation and evaluation

At the end of the FWRI surveillance period, a final report comprising cumulative FWRI surveillance statistics and a summary of reporting compliance by sentinel hospitals was generated and submitted for dissemination.

For internal evaluation purposes, we estimated three performance metrics: the proportion of action requests resolved, the proportion of sentinel sites achieving timely reporting and the process flow (the time between ONEISS data download and surveillance report creation).

## Results

**Fig. 2:**
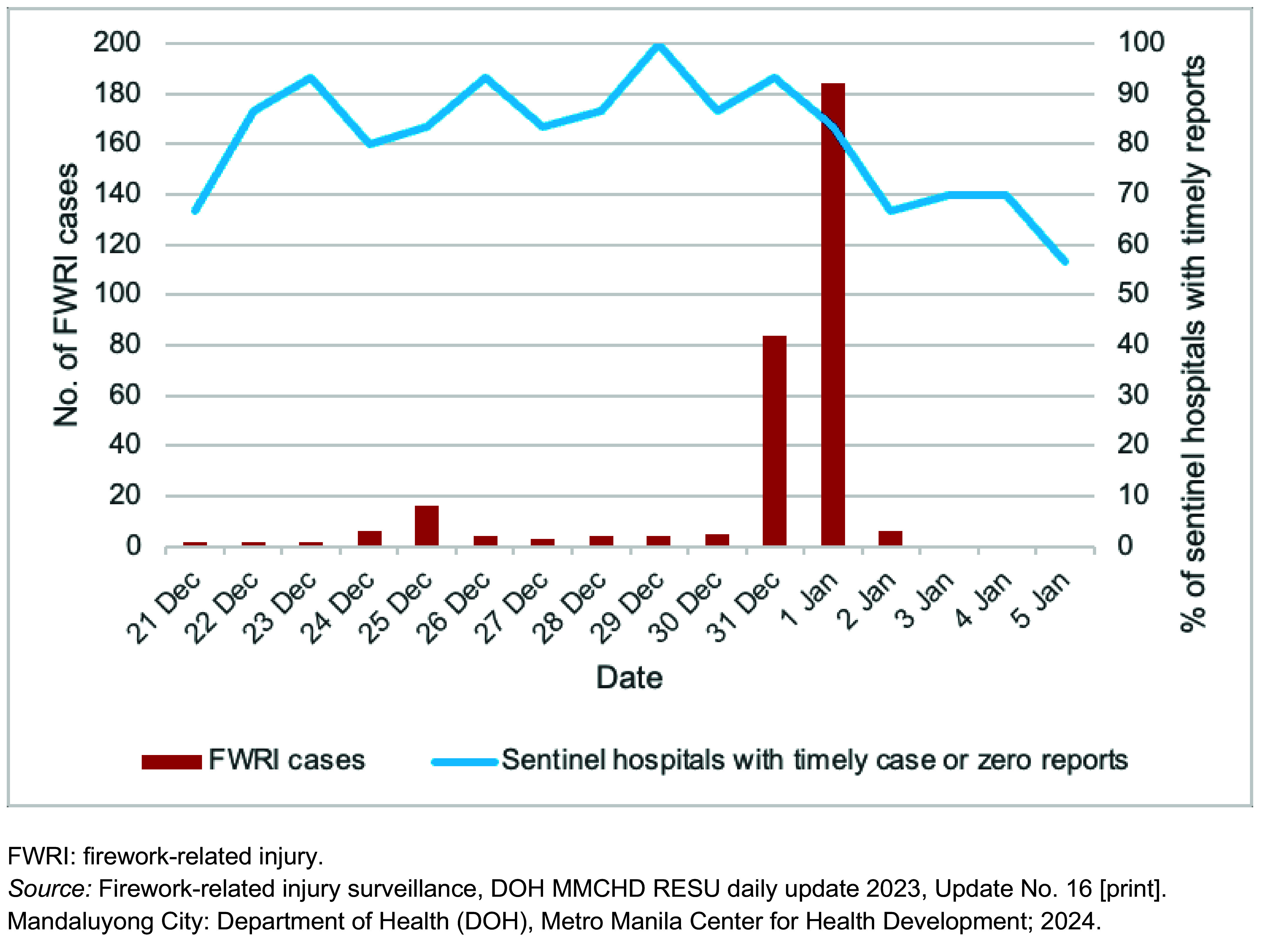
FWRI cases and sentinel hospitals with timely reports for each day of the FWRI surveillance period, Metro Manila, Philippines, 2023–2024

A total of 322 FWRI cases were captured by ONEISS during the 2023–2024 surveillance period. The number of FWRIs reported per day ranged from 0 to 6 cases, except on 25 and 31 December and 1 January when 16, 84 and 184 cases were reported, respectively (**Fig. 2**). A total of 77 action requests were coursed through RESU staff, all of which were posted in the VCS and then relayed to sentinel hospitals. Likewise, responses from sentinel hospitals to all 77 action requests were relayed to the DOH CO.

[**insert** Figure 2]

The proportion of sentinel sites that submitted timely reports for each day of the surveillance period ranged from 56.7% (*n* = 17/30) to 100% (*n* = 30/30), with a median of 83.3% (*n* = 25/30). On 11 consecutive days of the 16-day surveillance period, reporting was timely for at least 80% of sentinel hospitals (**Fig. 2**).

**Fig. 3:**
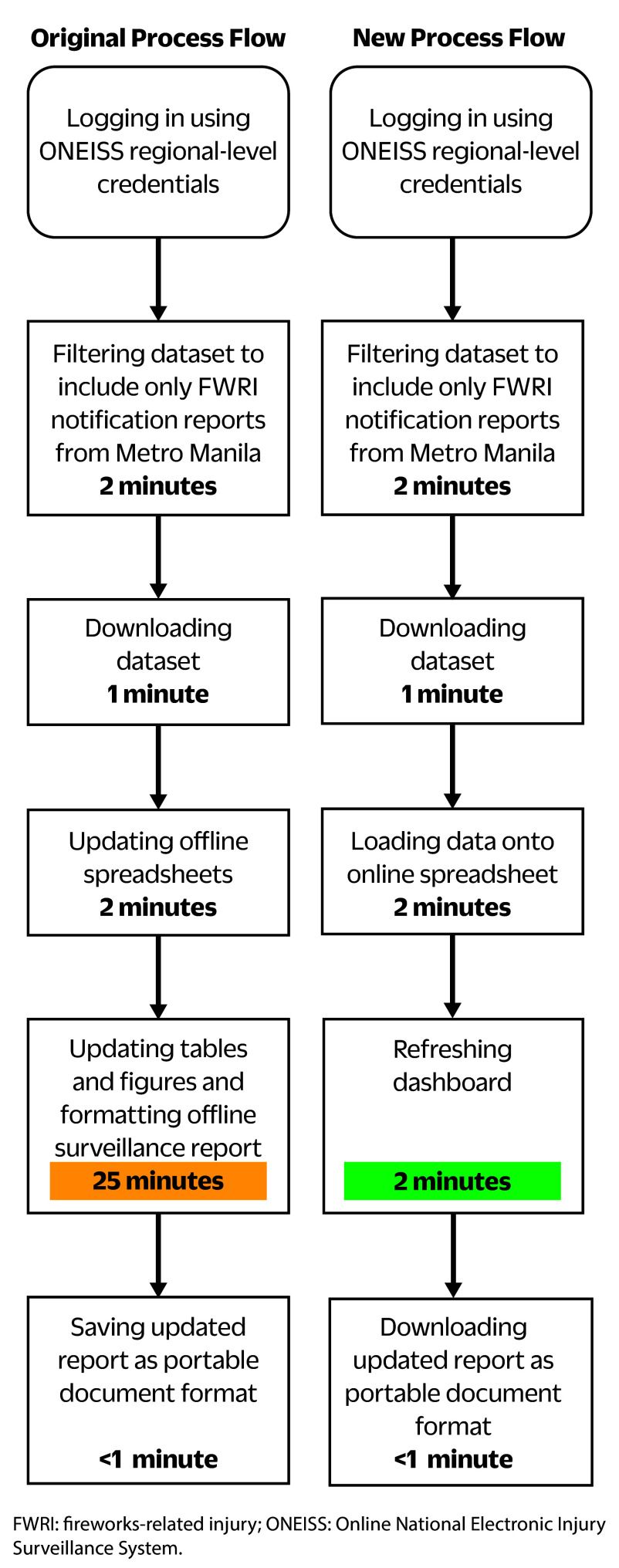
The original and new process flows for generating FWRI surveillance reports

The process flow for generating an FWRI surveillance report using the dashboard took approximately 8 minutes, which was faster than the original workflow of 31 minutes (**Fig. 3**).

[**insert** Figure 3]

## Discussion

The VCS and data dashboard contributed to improvements in FWRI surveillance process flows in Metro Manila. RESU staff were able to liaise continuously with the 30 sentinel hospitals and the DOH CO, resulting in high levels of timely reporting during the surveillance period. Data dashboards reduced the turnaround time for generating surveillance reports. While these tools are neither novel nor unique, to the best of our knowledge, their use at the regional level for FWRI surveillance has not previously been described.

The VCS delivered greater interconnectedness across the FWRI surveillance system; it facilitated connections between the DOH CO and sentinel hospitals and enabled RESU staff working in different locations across Metro Manila to coordinate their efforts. The VCS was most active during 03:00–05:00, especially on the nights of 24 and 31 December and 1 January, when the most FWRI cases were reported, indicating RESU staff’s high levels of commitment to their nightly coordination roles. There were several undocumented instances of sleep deprivation among staff who often had to work the following day. However, within their respective groupings, staff rotated nightly coordination duties to sustain the round-the-clock demands of FWRI surveillance whenever possible. The commitment shown by RESU staff, many of whom worked well beyond their normal hours, was a key factor behind the 100% response rate in processing requests from the DOH CO and the high levels of case validation. This dedication built not only a strong, lasting collaborative network of FWRI surveillance stakeholders, but also trust – which is essential for surveillance systems to thrive.

The human resource demands created by the VCS were to some extent offset by the dashboard, which relieved staff of the repetitive processes involved in manual data visualization. Dashboards are increasingly being used for process improvement, and in this setting demonstrably reduced workload by reducing turnaround time for generating reports. (*8*, *9*) Previous institutional experience in developing dashboards, in particular to support the COVID-19 response, helped with building the FWRI dashboard. (*10*) However, formal training on its use and management was not feasible due to time constraints. Staff responded by helping stakeholders familiarize themselves with the dashboard as needed, and one staff member took responsibility for its maintenance.

The main challenge encountered during implementation of the VCS and the dashboard was human resource constraints. Compared with other regions, which typically have fewer than four sentinel hospitals, FWRI surveillance in Metro Manila has a relatively greater complement of human resources and level of coordination covering all 30 sentinel hospitals. Even so, staff delegated to FWRI surveillance tasks were already either managing other surveillance systems or assigned as field staff in hospitals or local government units. Furthermore, most staff contracts ended on 31 December, affecting follow-up with sentinel hospitals for reporting and data validation during the last 5 days of FWRI surveillance. While the VCS and dashboard helped overcome this constraint, greater sustainability in FWRI surveillance requires solutions beyond process improvement, such as policy reforms allocating greater funding.

While we believe we have demonstrated that it is possible to operationalize regional dashboards, we acknowledge that further improvements in the VCS and the FWRI dashboard are needed to address evolving challenges and strengthen FWRI surveillance in Metro Manila. Data quality issues, such as missed and misclassified FWRIs, and underreporting at both sentinel and non-sentinel sites, have yet to be fully understood and addressed. Given that around 29% of all FWRIs in Metro Manila during the 2023–2024 surveillance period were reported by non-sentinel sites, the expansion of the surveillance network is key to future development, one where the VCS can play a role in facilitating stakeholder engagement and sustaining coordination. Additionally, the inclusion of visualizations of operational performance indicators (for example, the proportion of unreported and misclassified FWRI cases, and validated zero reports) in the dashboard would improve data quality and strengthen FWRI surveillance. There is also an opportunity to transform the FWRI dashboard into a public-facing dashboard, thereby helping raise public awareness of the risks of FWRIs.

### Conclusion

The implementation of FWRI surveillance is unique in Metro Manila primarily because of the concentration of sentinel sites. Significant improvements in turnaround time for generating surveillance reports were achieved through the adoption of innovative digital tools such as the VCS and data dashboards. These strategies can be scaled up to other surveillance systems and adopted by other organizations facing similar challenges. While continued use and development of such tools can contribute to stronger FWRI surveillance, it should be noted that policy reforms that increase funding would further strengthen FWRI surveillance and its sustainability in the Philippines.

## References

[1] DOH reports 85% decrease in fireworks injuries for 2021. Philippine News Agency; 1 January 2021. Available from: https://www.pna.gov.ph/articles/1126103, accessed 24 October 2025.

[2] Dela Peña K. Increase in firecracker injuries shows lessons never learned. Cebu Daily News; 31 December 2024. Available from: https://cebudailynews.inquirer.net/614901/increase-in-firecracker-injuries-shows-lessons-never-learned, accessed 22 October 2025.

[3] Cabato L. Fireworks-related injuries post 50% increase, says DOH. Inquirer.net; 6 January 2024. Available from: https://newsinfo.inquirer.net/1884763/fireworks-related-injuries-post−50-increase-says-doh, accessed 12 December 2024.

[4] Roca JB, de los Reyes VC, Racelis S, Deveraturda I, Sucaldito MN, Tayag E, et al. Fireworks-related injury surveillance in the Philippines: trends in 2010-2014. West Pac Surveill Response. 2015 Nov 11;6(4):1–6. 10.5365/wpsar.2015.6.1.014 26798555 PMC4712527

[5] Montemayor MT, Caliwan CL, Agoot L. DOH reports 443 firecrackers, stray bullet victims. Philippine News Agency; 2 January 2024. Available from: https://www.pna.gov.ph/articles/1216231, accessed 12 December 2024.

[6] Jaymalin M, Lazaro RE. Fireworks-related injuries up 39% – DOH. Philstar.com; 29 December 2022. Available from: https://www.philstar.com/nation/2022/12/29/2233881/fireworks-related-injuries−39-doh, accessed 12 December 2024.

[7] Rivera AS, Lam HY, Macalino JU. Epidemiology of injuries in the Philippines: an analysis of secondary data. Acta Med Philipp. 2018;52(2):180–6. 10.47895/amp.v52i2.442

[8] Wahi MM, Dukach N. Visualizing infection surveillance data for policymaking using open source dashboarding. Appl Clin Inform. 2019 May;10(3):534–42. 10.1055/s-0039-1693649 31340399 PMC6656571

[9] Weir BS, Vordtriede C, Lee JE, Metter EJ, Talbot LA. An interdisciplinary dashboard to streamline medication processing at patient discharge: a quality improvement initiative. Mil Med. 2023 Jul 22;188(7-8):usab526. 10.1093/milmed/usab526 34950952

[10] Ong KPS. Optimizing and automating aggregation and visualization of COVID-19 data in Metro Manila, Philippines, through the use of a free dashboard software: a case study. J Public Health Manag Pract. 2025 Nov-Dec 01;31(6):E361–7. 10.1097/PHH.0000000000002215 40779696

